# General population-based study on preferences towards end-of-life care in Southern Thailand: a cross-sectional survey

**DOI:** 10.1186/s12904-022-00926-3

**Published:** 2022-03-14

**Authors:** Aimorn Jiraphan, Jarurin Pitanupong

**Affiliations:** Department of Psychiatry, Faculty of Medicine, Prince of Songkla University, 90110, Hat Yai, Songkhla, Thailand

**Keywords:** Adult, End-of-life, Intervention, Older adult, Preference

## Abstract

**Background:**

End-of-life care preferences are potentially due to individual choice and feature variation due to culture and beliefs. This study aims to examine end-of-life care preferences and any associated factors, among the general Thai population. This could inform physicians in regards to how to optimize the quality of life for patients that are near the end of their lives.

**Methods:**

A cross-sectional study surveyed the general population in the Thai province of Songkhla; from January to April 2021. The questionnaires inquired about: 1) demographic information, 2) experiences with end-of-life care for their relatives, and 3) end-of-life care preferences. To determine end-of-life preferences, the data were analyzed using descriptive statistics. The data concerning patient demographics and end-of-life care preferences were compared using Fisher’s exact test.

**Results:**

The majority of the 1037 participants (67.6%) were female. The mean age among the adult and older adult groups were 40.9 ± 12.2, 70.0 ± 5.1, respectively. Half of them (48%) had an experience of observing someone die and 58% were satisfied with the care that their relatives had received. Most participants identified the following major end-of-life care preferences: having loved ones around (98.1%), being free from distressing symptoms (95.8%), receiving the full truth (95.0%), and having meaning in their lives (95.0%). There were no statistically significant differences in regards to end-of-life care preferences apart from being involved in treatment decisions**,** between adult and older adult groups.

**Conclusion:**

There was only one difference between the end-of-life preferences of the adult group versus the older adult group in regards to the topic of patient involvement in treatment decisions. Furthermore, receiving the full truth regarding their illness, being free from distressing symptoms, having loved ones around, and living with a sense of meaning were important end-of-life care preferences for both groups. Therefore, these should be taken into account when developing strategies towards improving patient life quality during their end-of-life period.

## Background

End-of-life (EoL) care interventions mainly focus on active pain control, withdrawal of futile life-sustaining treatment (LST), euthanasia, and physician-assisted suicide [1–3]. Patients’ ability to make decisions about their death process is generally seen as a vital component of ensuring a ‘good death’ [4]. Therefore, it is important to allow people to express, if they wish, their EoL preferences. There have been prior studies in regards to patients at advanced stages of terminal illness involved; these asked patients about what their wishes would be in certain scenarios. Nevertheless, these preferences potentially fluctuate over time and are associated with dynamic components of quality of life, such as functional status and psychological and spiritual dimensions [5]. Therefore, both patients and healthcare workers should be assisted to meet patients’ EoL preferences at the right time; whilst recognizing psychological defenses, and their evolution during the EoL process as well as appropriately managing conflicts or suffering in the patient-family unit [5, 6]. Additionally, promoting dignified dying should be an altruistic goal in palliative care because it helps direct interventions aimed at improving care at the end of life [7].

In regards to EoL preferences, euthanasia and other end-of-life decisions are acceptable to the large majority of the Western public [1–3]. In addition to having the capacity to say farewell and die with dignity and no pain, many westerners assign similar value to having control and their independence maintained in regards to having a ‘good death’ [8]. The acceptance of terminal sedation, increasing morphine dosage, and euthanasia were related to the wish to have a dignified death, and not wanting to burden relatives with terminal care [8]. However, there was a gender difference in regards to EoL preferences due to men being more willing than women are to agree with the concepts of physician-assisted suicide and/or euthanasia [9].

In the Asian region, a prior study involving Korean patients suffering cancer, their family caregivers, physicians, and the general Korean population identified that most physicians favored passive euthanasia, and that all people strongly favored the withdrawal of futile LST, and active pain control. In addition, ‘not being a burden to the family’ was positively related to preferences for active euthanasia [10]. However, attitudes towards EoL interventions may be different and significantly related with various attitudes towards death [11].

Furthermore, in accordance to a variety of studies, the factors associated with EoL preferences were: age, religion, education, economic status, occupation, self-satisfaction, current serious illness, hospitalization history, having caregivers or family, being a caregiver for a seriously ill patient, and the experience of someone close dying [12–15]. In regards to anxiety and thoughts about death, a study on age differences showed that both declined across people’s lifespan. In addition, having lower social support predicted higher levels of anxiety about death. Close relationships assisted emotion regulation functions, decreasing anxiety across the lifespan of participants and death-related thinking [16]. However, younger aged patients with advanced cancer desired and received more life-prolonging care than middle-aged and older patients. In addition, younger aged patients not desiring life-prolonging care were less likely to receive care consistent with their treatment preference versus middle-aged and older adults [17].

Nowadays, palliative care, an approach to improve the quality of life of patients facing a life-threatening illness and their families by relieving their symptoms and reducing suffering, including EoL care, is an important public healthcare field worldwide including the Asia-Pacific region [18], and Thailand [19, 20]. In 2019, a study found that Thai elderly patients and relatives from the central and northeast regions preferred to be: informed about essential issues, aware of the whole truth about their illness, free from uncomfortable symptoms, and able to pass away at their homes [12]. Likewise, a study conducted among Thai cancer patients and their relatives found that their main concern also was that they wished to pass away at their homes, having loved ones around, and having relief from uncomfortable symptoms such as shortness of breath and pain [13].

There is still work to be done until all patients receive an excellent level of care in regards to their EoL period as previous studies about this issue were conducted only in the central and northeastern regions of Thailand [12, 13]. There are limited data about the EoL care preferences of the general population, especially in regards to the southern region. Furthermore, several provinces in the southern region have a predominantly Muslim population; in contrast to other regions in Thailand where all provinces have majority Buddhist populations. In a religious context, a belief in life after death is central to the meaning and purpose of most Muslims’ lives. For followers of Islam, death marks the transition from one state of existence to the next. Islam teaches that life on earth is an examination and that the life to come is the eternal abode where one will reap the fruit of one’s endeavors on earth. Death is therefore not to be resisted or fought against, but rather something to be accepted as part of the overall divine plan [21]. Many Muslims believe that they are on this earth for a relatively short time and that, during this time, they are preparing themselves for eternal life after death. The Islamic belief about death has a positive meaning, therefore this can help Muslim patients to cope better near the time of their death [22]. Therefore, southern peoples’ EoL care preferences may be different. Additionally, Songkhla is the tenth most populous province in Thailand and a prominent province in Southern Thailand with over 1.4 million residents across a total area of approximately 2855 mile^2^ (7394 km^2^). Moreover, approximately 33.2% of the province of Songkhla’s population are Muslim. Thus, it may be a good representation of a southern province, which has a mixture of cultures and beliefs. To our knowledge, no study on EoL care preferences has been conducted in the southern region of Thailand over the past decade apart from a recent survey in regards to cancer patients [14]. Therefore, this study aims were to evaluate Thai peoples’ preferences about EoL care interventions for themselves and their relatives and to evaluate factors associated with these preferences; in regards to all age groups. Furthermore, to provide knowledge that may be useful towards developing and establishing effective EoL caring processes via a variety of psychosocial support frameworks.

## Methods

After being approved by the Ethics Committee of the Faculty of Medicine, Prince of Songkla University (REC: 63–200–3-1), this cross-sectional study was conducted by a self-administrated questionnaire by the adult and older adult general population in the Thai province of Songkhla; from January to April 2021. To be included in the study, participants must have been: part of the general population, aged 20 years or above, able to understand and use the Thai language well, in agreement to participate in the study, and able to complete all questionnaires. Participants were excluded if they had the following: progressive neurological disease (e.g., dementia, Parkinson’s, motor neuron disease), advanced heart disease, chronic lung disease, terminal illness (e.g., advanced cancer) or pregnancy. In addition, participants were excluded if they lacked the mental capacity to complete all of the questionnaires, or if they decided to withdraw from the study.

In respect to the sample size calculation, the literature review suggested that the proportion of patients that agreed with each item of adult EoL care preference had to be at least 50% [12] and that 29.2% are older adults [13]. In regards to getting the maximum sample size and a 5.0% margin error, the function n.for. the survey from the R program was used for the calculation of the sample size for a survey with cluster sampling (given delta = 0.05, design effect = 1.5, and alpha = 0.05). Then, the sample size required was 592 for adults (aged 20–64 years) and 484 for older adults (age older than 65 years). The sample size in each district was based on the number in regards to each population (population proportionate to size).

The authors performed quota sampling according to the number of residents in the district and distributed this to account for both age groups. The authors sent a letter requesting permission from the Songkhla provincial public health office to collect data and perform the research. Then the researcher informed the village health volunteers, who provided research assistance, about the study’s rationale and objectives and trained them on the study procedures based on a manual outlining the fieldwork guidelines that the authors developed for this study. The village health volunteers recruited participants via convenience sampling according to the sample size number for each age group, handed them an information sheet including the study rationale and the time slot to complete the self-administered questionnaires. The participants had at least 10–15 min to consider whether to collaborate in the study or not. If they wished to collaborate, they were all asked to sign the informed-consent forms and were invited to a private zone for the self-administrated questionnaires taking 15–30 min. The village health volunteers observed the reaction of the participants and informed them that they could stop the self-administered questionnaires at any time if they felt uneasy, distressed, or were no longer willing to participate.

### Measures

The questionnaires were reviewed by 5 psychiatrists (one of them practiced in the Palliative Care Unit of our hospital), and content validity was performed yielding a content validity index (CVI) score of 0.8. A pilot study was conducted with 25 participants, and the Cronbach’s alpha was 0.8. The questionnaire was composed of 3 parts:

1. Personal and demographic information inquiry consisting of questions related to age, gender, religion, marital status, education, income, number of household members, physical and psychiatric illness, history of hospital admission, history of substance use, and perception concerning their satisfaction with their health and life.
2. Inquiry regarding experiences with EoL care for their relatives, which consisted of 10 questions on experiences related to seeing relatives die and being an EoL caregiver. Each item was rated as ‘yes’ or ‘no’; the attitude toward experiences with the death of a relative was rated as ‘satisfied’, ‘unsatisfied’, or ‘neither satisfied nor unsatisfied’, meanwhile the attitude towards their relatives being remembered after their death was rated as ‘agree’, ‘disagree’, or ‘neither agree nor disagree’ [13].
3. Inquiry on EoL care preferences focusing on the sort of EoL care they would like for themselves, which consisted of 2 categories (total 15 items) [11–13].
  3.1 Importance of EoL care comprised the 12 following items: receiving the full truth about their illnesses, disclosing the truth about their illnesses to family members, being involved in treatment decisions, receiving both physical and psychological treatment, receiving relief from distressing symptoms, not being a burden to their family, feeling that life is meaningful, participating in and performing religious rituals, completing unfinished business and being prepared to die, have loved ones around when needed, be mentally aware at the last hour, and passing away at home
  3.2 EoL care preferences consisted of the 3 following items: withholding of futile LST, the administration of active pain control, and performing active euthanasia.

The scores of all the 15 questions ranged from 1 to 5 (strongly agree, agree, disagree, strongly disagree, and neither agree nor disagree). The responses of ‘strongly agree’ and ‘agree’ were classified as indicating approval for the intervention [12, 13]. Furthermore, the participants were asked to rate the 3 most important components among the 15 items.

### Statistical methods

Descriptive statistics such as mean, proportion, and standard deviation (SD) were calculated. The data relating to patient demographics and EoL care preferences were compared using Fisher’s exact test. The statistical significance was defined as a *p*-value of less than 0.05.

## Results

### Demographic characteristics

From January to April 2021, 1037 participants collaborated in the survey by completing the constructed questionnaires. The response rate was 96.4%. The majority of them were female (67.6%), married (68.2%), and Buddhist (67.7%). Their mean age was 54.0 ± 17.4 years. The mean age between the adult and the older adult group were 40.9 ± 12.2, 70.0 ± 5.1, respectively. In addition, most participants had no physical illness (73.5%), never had a history of hospitalization (76.8%), and were satisfied with their life (84.2%) and health (60.8%) (Table 1). The most common physical diseases were hypertension (11.9%), diabetes mellitus (6.3%), and dyslipidemia (2.1%).

**Table 1:** Demographic characteristics (*N* = 1037)

Demographic characteristics	Total (***N*** = 1037)	Number (%)	
		Adult (***n*** = 570)	Older adult (***n*** = 467)
Gender			
Male	333 (32.1)	150 (26.3)	183 (39.2)
Female	701 (67.6)	418 (73.3)	283 (60.6)
No answer	3 (0.3)	2 (0.4)	1 (0.2)
Religion			
Buddhism	702 (67.7)	386 (67.7)	316 (67.7)
Islam, Christianity	117 (11.3)	84 (14.7)	33 (7.1)
No answer	218 (21.0)	100 (17.5)	118 (25.3)
Marital status			
Single/ divorced	310 (29.9)	200 (35.1)	110 (23.6)
Married	707 (68.2)	356 (62.5)	351 (75.2)
No answer	20 (1.9)	14 (2.5)	6 (1.3)
Education			
Primary school and below	229 (22.1)	42 (7.4)	187 (40.0)
Secondary school	159 (15.3)	100 (17.5)	59 (12.6)
Diploma	159 (15.3)	92 (16.1)	67 (14.3)
Bachelor’s degree and above	482 (46.5)	332 (58.2)	150 (32.1)
No answer	8 (0.8)	4 (0.7)	4 (0.9)
Occupation			
Civil servant, government employee	177 (17.1)	99 (17.4)	78 (16.7)
Entrepreneur	48 (4.6)	18 (3.2)	30 (6.4)
Technical or service staff	112 (10.8)	81 (14.2)	31 (6.6)
Agriculturist/fisherman	284 (27.4)	115 (20.2)	169 (36.2)
Craftsman	264 (25.5)	192 (33.7)	72 (15.4)
Student/unemployed	144 (13.9)	61 (10.7)	83 (17.8)
No answer	8 (0.8)	4 (0.7)	4 (0.9)
Income (Baht/month)			
< 10,000	437 (42.1)	212 (37.2)	225 (48.2)
10,001 – 20,000	298 (28.7)	206 (36.1)	92 (19.7)
> 20,000	291 (28.1)	148 (26.0)	143 (30.6)
No answer	11 (1.1)	4 (0.7	7 (1.5)
Physical illness			
No	762 (73.5)	487 (85.4)	275 (58.9)
Yes	226 (21.8)	52 (9.1)	174 (37.3)
No answer	49 (4.7)	31 (5.4)	18 (3.9)
Number of household members			
Alone	50 (4.8)	37 (6.5)	13 (2.8)
Less than 3	409 (39.4)	225 (39.5)	184 (39.4)
More than 3	572 (55.2)	306 (53.7)	266 (57.0)
No answer	6 (0.6)	2 (0.4)	4 (0.9)
History of admission			
Yes	235 (22.7)	94 (16.5)	141 (30.2)
No	796 (76.8)	475 (83.3)	321 (68.7)
No answer	6 (0.6)	1 (0.2)	5 (1.1)
Satisfaction with own health			
Excellent	196 (18.9)	135 (23.7)	61 (13.1)
Good	434 (41.9)	272 (47.7)	162 (34.7)
Fair/Poor	406 (39.2)	163 (28.6)	243 (52.0)
No answer	1 (0.1)	0 (0.0)	1 (0.2)
Satisfaction with life			
Satisfied	873 (84.2)	494 (86.7)	379 (81.2)
Unsatisfied	50 (4.8)	20 (3.5)	30 (6.4)
No answer	114 (11.0)	56 (9.8)	58 (12.4)

### Experiences and attitude in regards to end-of-life care for relatives

There were 498 participants (48%) who reported having had the experience of seeing a loved one die, and 46.9% of them had had the role of an EoL caregiver with statistically significant differences between adult and older adult groups in regards to both experiences (*p*-value<0.001). The majority of them were satisfied with the EoL care that their relatives received and with the knowledge that they were being remembered after their death (58.0%) (Table 2).

**Table 2:** Experiences with and attitude towards the end-of-life care for their relatives (*N* = 498)

Type of EoL care	Total (***n*** = 498)	number (%)		Chi^**2**^ ***P***-value
		Adult	Older adult	
		(***n*** = 220)	(***n*** = 278)	
Having the experience of seeing a patient who is at the end of life				< 0.001
Yes	498 (48.0)	220 (38.7)	278 (59.7)	
No	537 (51.8)	349 (61.3)	188 (40.3)	
Having the experience of caring for patient who is at the end of life				< 0.001
Yes	486 (46.9)	205 (36.2)	281 (60.6)	
No	545 (52.6)	362 (63.8)	183 (39.4)	
Being hospitalization				0.1
Yes	396 (79.5)	177 (89.4)	219 (83.6)	
No	64 (12.9)	21 (10.6)	43 (16.4)	
Passing away at home among family				0.407
Yes	309 (62.0)	128 (65)	181 (69.1)	
No	150 (30.1)	69 (35)	81 (30.9)	
Endotracheal intubation				0.643
Yes	287 (57.6)	121 (60.8)	166 (63.4)	
No	174 (34.9)	78 (39.2)	96 (36.6)	
Cardiopulmonary resuscitation				0.916
Yes	146 (29.3)	62 (31.2)	84 (32.1)	
No	315 (63.3)	137 (68.8)	178 (67.9)	
Intravenous infusion				0.953
Yes	204 (41.0)	87 (43.9)	117 (44.7)	
No	256 (51.4)	111 (56.1)	145 (55.3)	
Use nasogastric tube or enterostomy feeding				0.182
Yes	162 (32.5)	77 (38.9)	85 (32.4)	
No	298 (59.8)	121 (61.1)	177 (67.6)	
Use of analgesic agents				0.004
Yes	216 (43.4)	109 (55.1)	107 (41.0)	
No	243 (48.8)	89 (44.9)	154 (59.0)	
Attitude toward being remembered after death				0.311
Yes	289 (58.0)	120 (58.3)	169 (64.5)	
Neutral	133 (26.7)	62 (30.1)	71 (27.1)	
No	46 (9.2)	24 (11.7)	22 (8.4)	

There were missing values for some variables

### End-of-life care preferences

Concerning the importance of EoL care, most participants identified five major care components as the most important ones: having loved ones around when needed (98.1%), receiving both physical and psychological treatment (96.3%), receiving relief from distressing symptoms such as shortness of breath and pain (95.8%), receiving the full truth about their illnesses (95.0%), and having a sense of meaning in life (95.0%). However, passing away at home was rated as the least important component (84.6%) (Table 3).

**Table 3:** End-of-life care preferences (*n* = 1037)

	***Total (n = 1037)***	number (%)		Chi^**2**^ ***P***-value
		adult	Older adult	
		(***n*** = 570)	(***n*** = 467)	
Receiving the full truth regarding their illness				0.489
No opinion	2 (0.2)	1 (0.2)	1 (0.2)	
Disagree	49 (4.7)	31 (5.4)	18 (3.9)	
Agree	985 (95.0)	538 (94.4)	447 (95.9)	
Disclosing the full truth regarding their illness to family members				0.329
No opinion	1 (0.1)	0 (0.0)	1 (0.2)	
Disagree	57 (5.5)	35 (6.2)	22 (4.7)	
Agree	978 (94.3)	534 (93.8)	444 (95.1)	
Having loved ones around when needed				0.95
Disagree	17 (1.6)	10 (1.8)	7 (1.5)	
Agree	1017 (98.1)	560 (98.2)	457 (98.5)	
Not being a physical or psychological burden to the family				0.629
No opinion	4 (0.4)	3 (0.5)	1 (0.2)	
Disagree	87 (8.4)	50 (8.8)	37 (7.9)	
Agree	942 (90.8)	514 (90.7)	428 (91.8)	
Completing unfinished business; preparing to die				0.947
No opinion	13 (1.3)	7 (1.2)	6 (1.3)	
Disagree	40 (3.9)	23 (4.0)	17 (3.6)	
Agree	983 (94.8)	540 (94.7)	443 (95.1)	
Having the sense of being meaningful in life				0.05
No opinion	21 (2.0)	16 (2.8)	5 (1.1)	
Disagree	29 (2.8)	12 (2.1)	17 (3.7)	
Agree	985 (95.0)	542 (95.1)	443 (95.3)	
Being free from distressing symptoms such as pain and shortness of breath				0.368
No opinion	6 (0.6)	2 (0.4)	4 (0.9)	
Disagree	34 (3.3)	16 (2.8)	18 (3.9)	
Agree	993 (95.8)	548 (96.8)	445 (95.3)	
Receiving both physical and psychological treatment				0.809
Disagree	36 (3.5)	21 (3.7)	15 (3.2)	
Agree	999 (96.3)	548 (96.3)	451 (96.8)	
Performing or participating in religious rituals				0.186
No opinion	41 (4.0)	19 (3.3)	22 (4.7)	
Disagree	54 (5.2)	35 (6.1)	19 (4.1)	
Agree	942 (90.8)	516 (90.5)	426 (91.2)	
Being involved in treatment decisions				0.005
No opinion	47 (4.5)	19 (3.3)	28 (6.0)	
Disagree	58 (5.6)	23 (4.0)	35 (7.5)	
Agree	930 (89.7)	526 (92.6)	404 (86.5)	
Being mentally aware at the last hour of life				0.427
No opinion	32 (3.1)	14 (2.5)	18 (3.9)	
Disagree	61 (5.9)	34 (6.0)	27 (5.8)	
Agree	941 (90.7)	521 (91.6)	420 (90.3)	
Passing away at home				0.583
No opinion	58 (5.6)	29 (5.1)	29 (6.2)	
Disagree	98 (9.5)	51 (9.0)	47 (10.1)	
Agree	877 (84.6)	488 (85.9)	389 (83.7)	
Withhold of futile life-sustaining treatment				0.447
No opinion	65 (6.3)	31 (5.5)	34 (7.3)	
Disagree	60 (5.8)	32 (5.6)	28 (6.0)	
Agree	908 (87.6)	505 (88.9)	403 (86.7)	
Active pain control				0.09
No opinion	24 (2.3)	16 (2.8)	8 (1.7)	
Disagree	212 (20.4)	104 (18.3)	108 (23.2)	
Agree	797 (76.9)	448 (78.9)	349 (75.1)	
Active euthanasia				0.128
No opinion	66 (6.4)	30 (5.3)	36 (7.7)	
Disagree	113 (10.9)	69 (12.2)	44 (9.5)	
Agree	853 (82.3)	468 (82.5)	385 (82.8)	

There were missing values for some variables

Regarding EoL care preferences, most participants reported a high level of preference for withholding of futile LST (87.6%) (Table 3). Moreover, the 3 most important reported components regarding EoL care wishes were: receiving the full truth about their illnesses, not being a burden to the family, and participating or performing in religious rituals.

Additionally, comparing between adult and older adult groups, there was no statistically significant difference in EoL care preference in regards to the importance of EoL care and EoL care preferences with the exception of being involved in treatment decisions (Odd ratio 1.98; 95%CI 1.15–3.41, *p* = 0.013).

### Influence of demographic factors, experiences related to EoL care for relatives on preferences for EoL care

Majority of responses were in agreement with one another, so no significant differences exist in age groups on EoL care preferences. Additionally, it was an attempt to identify the factors related with EoL care preferences but no statistically significant association was found in the multivariate analysis.

## Discussion

This survey was the first to study EoL care preferences in the general adult and older adult population in Southern Thailand. In this study, most participants identified, as important, five major EoL care preferences: receiving the full truth about their illnesses, receiving both physical and psychological treatment, being relieved from distressing symptoms such as pain and shortness of breath, having loved ones around when needed, and having the sense of having had a meaningful life. Moreover, the top 3 most important components regarding EoL care wishes were: receiving the full truth about their illnesses, not being a burden to the family, and participating or performing in religious rituals. These findings were concordant with earlier reports from the USA [23] together with a study regarding Thai elderly people [12], and Thai palliative cancer patients [14]. A reason for this may be due to most participants in this study being older adults with a mean age of 54 years. Furthermore, receiving the full truth about one’s illness, being relieved from distressing symptoms, having the sense of having meaning in life or participating in religion or such values, and having loved ones around when needed may all be universal basic human needs during the EoL period. Therefore, there were no age or cultural differences.

Receiving the full truth about one’s illness was one of the most important components of their EoL care. This highlights that autonomy was a key area of concern for everyone in regards to the EoL period. In regards to comparing the adult group with the older adult group, this study identified that the adult group wanted to be involved in treatment decisions statistically significantly more than the older adult group. A reason for this may be due to higher levels of death anxiety, or the need for more life-prolonging care among the younger group [17], that may make them more likely to want to have a level of power to control such situation. Therefore, the healthcare providers should be concerned about patients’ autonomy, be more interactive and let the patients take part of the treatment care process including EoL procedures. Moreover, design to receive care both in regards to their ‘disease’ and to their sense of ‘humanity’ should be highlighted.

The earlier reports from Switzerland revealed that the relief of suffering from specific physical and non-physical symptoms by competent healthcare professionals was mentioned by the majority of the general population [24]. However, a prior study identified that decision-making in regards to medical treatments during the EoL period is inadequate. To reduce decisional conflict, patients and their families need more support to clarify their values and to ensure that their preferences are grounded in an adequate understanding of their illness and treatment options [25]. Therefore, healthcare professionals should provide good communication, acceptance, compassion and create an empathetic atmosphere to enhance the patient’s cooperation, trust, dignity, and peace.

Additionally, being free from distressing symptoms, pain and shortness of breath was the topmost concern for participants. This issue might be a universal need among all people and patients in regards to quality of life when receiving EoL care. Moreover, this finding was similar to a prior study among Thai palliative cancer patients [14] highlighting that pain management was still an unsolved issue needing more attention from physicians tasked with identifying and alleviating pain and other distressing symptoms [26]. Therefore, patients’ anxiety about the management of their suffering, by the healthcare system, during their EoL period should be tackled.

Furthermore, having the sense of having meaning in life was a major EoL preference in the context of having had a nice life and a good death. The increase in patients’ perception of dignity and meaning in life can help patients in preparing for death [27]. Additionally, focusing on completing activities related to their lives and prioritizing their ability to do this as well as intending to avoid leaving any unfinished business to their loved ones were important issues. Therefore, organizing their affairs also meant that when reaching their end of life, it provided them peace of mind to know that their family members were aware of their preferences. Families should also be encouraged to organize affairs and have everything settled for the loved ones of the person dying [24].

In regards to having a good death, the participants desired to be able to have loved ones around them when they required this. Connection and support with loved ones is important in any uncertain situation. In a prior study, EoL care was related with the desire of having close friends and family around until the end; by maintaining trusting relationships and not being alone [24]. For people with a life-limiting illness, it was significant to maintain autonomy for as long as possible including maintaining social relationships, being engaged in everyday life, having the ability to prepare for dying and death while living life as normally as possible.

This included the willingness to not be left alone at the time of death, something that should be factored in the approach taken by palliative healthcare teams [28–32]. Therefore, physicians should consider the importance of providing a sense of love and peace for patients during their EoL periods. However, our study found that in regards to the EoL preference of having loved ones around when needed but not being a physical or psychological burden to them, may contribute to a feeling of ambivalence towards their family. In regards to the nature of Thai people, even though they need someone around, they also often tend to be considerate, guilty, and not daring to express their inner desires in a frank manner. Therefore, changing the meaning of the family burden to a positive one can help a person lessen their feelings of ambivalence and empower them to be able to tell their feelings honestly.

Additionally, in this study, passing away at home was rated as the least important component, indicating that attitudes about the place of death may have changed versus common attitudes in the past. Potential explanations could be that when someone chooses to pass away at home, this may increase the physical and psychological burden to family members. Furthermore, for some patients, dying with a sense of meaning and being remembered after death might be more significant than the place of death [14, 33]. Choosing to die in a hospital may be preferable for the caregivers of palliative patients [34] because of the difficulties associated with palliative home care due to factors such as poor family networks and/or low levels of home care support from the public health system [35, 36]. However, there was a higher level of preferences for dying at home when their caregivers and the family physician made home visits [37]. Most prior studies, on people’s preferences about the places of care and death, have been cross-sectional, assessing preferences at a specific point in time and usually during hospital admission [38].

The few longitudinal investigations conducted, suggest that choices change over time, shifting slightly from home towards hospital death [39]. Furthermore, people adjust their choices and renegotiate their priorities, and these five aspects seem particularly influential in shaping patients’ and carers’preferences regarding their place of death: symptoms and physical management, existential perspectives, quality of life, informal care resources, and patients’ experiences of services and environments, [39]. Additionally, many people dying in institutions had unmet needs in regards to symptom amelioration, emotional support, physician communication, and being treated with respect. The patients’caregivers who received care at home with hospice services were more likely to report that it was a more preferable situation [40]. Accordingly, paying attention to the desires of patients in regards to their wish to receive any type of EoL care as well as to have their definition of meaningful death respected, is crucial for both healthcare providers and caregivers.

In regards to our study, most of the participants have agreed to the concept of active euthanasia in both adult and older adult groups. Nevertheless, active euthanasia and physician-assisted suicide are illegal in Thailand. Yet, under Section 12 of the Public Health Act, patients in the terminal stage of their life have the right to refuse treatment. In addition, patients can prepare a “living will”, which expresses their desire to reject treatment, in advance [41]. Thanks to this law, patients can pass away painlessly and peacefully. In Thailand, euthanasia has been the subject of relevant discussions and it is suggested that further discussions are needed in the future, as the country is fast becoming an ageing society.

Finally, this cross-sectional study took place during the Covid-19 global pandemic. In Songkhla, there are presently at least 66,000 confirmed cases of COVID-19 and over 300 deaths due to the virus. Thai people regularly listen to the most recent updates on the number of cases and deaths from COVID-19. Furthermore, a prior study on the COVID-19 pandemic and attitudes toward death have showed that the participants featured attitudes of fear and avoidance [42]. This may alter the attitude of participants in this study. They might feel strongly affected by personal loss, grief, and a paralyzing fear of death. However, on the other hand, they might have been more prepared, having considered their end-of-life care preferences in the context that their death is near.

To our knowledge, this study was the only survey conducted on this topic among the general adult and older adult population in Southern Thailand during the past decade. However, it employed a cross-sectional survey and self-administered questionnaires, increasing the risk of bias. In addition, the study was quantitative, and the sample size was restricted to the general population of the lower part of Southern Thailand. Thus, it might not be used to draw general conclusions about the Thai population on a national level. It is recommended that multi-center studies are conducted that include population from other areas within Thailand. Furthermore, those future studies should employ more qualitative, in-depth methods for specific disorders. However, only 11.3% of the participants enrolled in this study identified with Islamic or Christian beliefs, and this discrepancy may form the basis for another limitation in this study as its aim was to demonstrate differences in regards to EoL care preferences in the context of a mixed culture community.

## Conclusion

There was only one difference between the end-of-life preferences of the adult group versus the older adult group in regards to the topic of patient involvement in treatment decisions. Furthermore, receiving the full truth regarding their illness, being free from distressing symptoms, not incurring any physical and/or psychological burden to their families, performing or participating in religious rituals, having meaning in life, and having loved ones around when needed were the most important factors indicated in regards to potentially improving patients’ quality of life during their EoL periods.

## Data Availability

The datasets used and/or analyzed during the current study can be made available by the corresponding author upon reasonable request.

## Abbreviations

EoL: End-of-life
LST: Life-sustaining treatment
